# Editorial: Importance of Root Symbiomes for Plant Nutrition: New Insights, Perspectives and Future Challenges

**DOI:** 10.3389/fpls.2020.00594

**Published:** 2020-05-13

**Authors:** Kevin Garcia, Heike Bücking, Sabine D. Zimmermann

**Affiliations:** ^1^Department of Crop and Soil Sciences, North Carolina State University, Raleigh, NC, United States; ^2^Department of Biology and Microbiology, South Dakota State University, Brookings, SD, United States; ^3^BPMP, Univ Montpellier, CNRS, INRAE, Institut Agro, Montpellier, France

**Keywords:** biological nitrogen fixation, mycorrhizal symbiosis, plant growth promoting rhizobacteria, plant nutrition, root microbiome

Plants interact with a plethora of soil microbes that help them to acquire nutritional resources, to be protected against pathogens, and to face challenging and fluctuating external conditions. Understanding how the microbiota of roots and rhizospheres is shaped and conserved by host plants, and how it changes in response to genetic and environmental pressures, is crucial for the preservation of natural ecosystems and to harness its potential for the development of novel strategies in agroecosystems. This Research Topic presents a series of articles that summarizes the latest research updates on the impact of the plant microbiota and its specific symbionts [i.e., arbuscular mycorrhizal (AM) and ectomycorrhizal (ECM) fungi, nitrogen-fixing rhizobia, and plant growth-promoting rhizobacteria (PGPR)] on plant performance and resilience, and the external factors that influence plant microbiota assembly.

Novel next generation sequencing technologies and analytical platforms provide us with more insights into the plant microbiome. In this Research Topic, Ma et al. discussed the development and potential use of single-cell RNA-sequencing technology in the area of plant microbiomes. Although still challenging to apply routinely, it opens the way toward the discovery of very specific and localized functions of bacterial communities in plant microbiota. Multiple studies have shown that the microbiome composition differs among cultivars of a given plant species, such as corn (Walters et al., 2018), cotton (Wei et al., 2019), or grape (Mezzasalma et al., 2018). Using three switchgrass cultivars grown under various conditions (i.e., monoculture, intraspecific, or interspecific mixtures), Revillini et al. showed that plant diversity influences the structure of AM fungal and bacterial communities in the root rhizosphere, but that nitrogen fertilization only affects the composition of the AM community but not of the rhizobacterial community. These findings highlight the importance of adapted cultivar and management practices in agricultural settings to maintain optimal microbiomes. Although it is described that herbivores can also influence the leaf microbiota composition (Humphrey and Whiteman, 2020), their impact on root microbiota assembly, and particularly on AM fungi, is still unclear. In this Research Topic, Wilkinson et al. demonstrated that the inoculation with aphids does not alter the AM colonization and community composition in barley, but that the formation of fungal vesicles and the relative abundance of some fungal species in these communities is affected.

The root microbiome also has a tremendous influence on forest diversity, evolution, and dynamic, defining a complex “ecosystem microbiome” (Baldrian, 2016). ECM fungi play a significant role in Northern hemisphere forest ecosystems (Becquer et al., 2019). Nagati et al. investigated the community assembly of ECM fungi on balsam fir seedlings in two biotically different environments and their potential role in the spread of the species. Using path analysis, they identified significant correlations between stand type, sapling growth, foliar nitrogen content and understory vegetation on community composition, and presented an exciting effort toward a better understanding of the microbial mechanisms underlying forest succession. Although significant progress has been made in the identification and characterization of plant and fungal transport proteins (Garcia et al., 2016; Plassard et al., 2019) in the ECM symbiosis, there are many key players that are still missing, particularly for micronutrients such as zinc. In this Research Topic, Coninx et al. revealed the dual function of the transporter SlZRT2 from the ECM fungus *Suillus luteus* in zinc acquisition from the external medium and its redistribution in fungal hyphae in response to zinc availability (Ruytinx et al., 2020).

Biological nitrogen fixation within root nodules is a tremendous advantage conferred by specific soil rhizobia bacteria to certain plant families, allowing the acquisition of atmospheric nitrogen (Griesmann et al., 2018). Purple acid phosphatase is a class of proteins involved in phosphorus acquisition and utilization, particularly under limiting conditions. Using transcriptomic and reverse genetics approaches, Wang et al. described a novel purple acid phosphatase of soybean (GmPAP12) required for nodule formation and nitrogen fixation, reinforcing the importance of plant phosphorus nutrition in biological nitrogen fixation. The impact of an inoculation with nitrogen fixing bacteria on agroecosystems and forest ecosystems for restoration purposes highly depends on various soil amendments. Egamberdieva et al. studied the effect of two maize biochars on biological nitrogen fixation in chickpea under drought and irrigated conditions, and observed an improved growth, nitrogen uptake, and symbiotic performance under both conditions. Zhang et al. investigated the effect of different nitrogen sources on the colonization with rhizobia in two major forest restoration species (*Robinia pseudoacacia* and *Lupinus latifolius*), and found a synergistic response between nitrogen supply and bacterial inoculation. *Parasponia* (Cannabaceae) is the only non-legume lineage that has evolved the ability to develop symbiotic nodules with nitrogen-fixing rhizobia (Op den Camp et al., 2011). In legumes and actinorhizal plants, soil nitrate acts as a negative regulator of nodule development (Ferguson et al., 2019). Using *Parasponia andersonii* as a model plant, Dupin et al. investigated the impact of increasing ammonium nitrate concentrations on tissue weight, nodule formation, and nodule number. The authors observed that *P. andersonii* displayed a reduction of symbiotic nodules after external nitrogen application, or even an elimination at high concentrations, indicating the presence of a similar autoregulation mechanism than has been described for legume and actinorhizal plants.

PGPR are soil bacteria that infect roots and confer various benefits to the colonized plants, such as improved growth, resistance to biotic and abiotic stress, or nutrient acquisition (Backer et al., 2018). Gupta and Pandey isolated two new bacterial strains from the rhizosphere of *Allium sativum* exhibiting ACC deaminase activity, as well as the production of indole acetic acid, siderophore, ammonia, and hydrogen cyanide. These bacteria, identified by 16S rRNA sequencing as *Aneurinibacillus aneurinilyticus* and *Paenibacillus* sp., are also able to solubilize insoluble forms of phosphate and zinc. The combined application of these two strains on *Phaseolus vulgaris* was able to improve root length, shoot length, and root and shoot biomass under salt stress conditions. Another study from Mukherjee et al. described the isolation of a novel *Halomonas* species from the rhizosphere of true mangrove *Avicennia marina*. Inoculated with rice, this PGPR led to growth promotion under combined salt and arsenic stresses, through the direct involvement of bacterial exopolysaccharide. Both studies highlight the importance of natural diversity screenings to isolate novel plant beneficial microbes that can be used in agroecosystems. Finally, Chang et al. reviewed the current research on cycad-cyanobacteria symbioses. Due to the slow growth of cycads, these palmlike woody plants are under-researched, and the authors revealed important gaps in our knowledge about these interactions which should be addressed by the functional comparison with other cyanobacterial symbioses.

Overall, our knowledge on the diversity, composition, and impact of plant microbiota is still limited. It is crucial to continue investigating the molecular biology, physiology, and ecology of mutualistic interactions to isolate novel symbionts and microbial properties that improve plant performance and resilience. We hope that the work presented in this Research Topic will help to both consolidate the field of plant-microbe symbioses and bring light on the importance of understanding and preserving the plant microbiota in natural and agroecosystems.

## Author Contributions

KG wrote the editorial, which was revised, proofed, and accepted by all the authors.

## Conflict of Interest

The authors declare that the research was conducted in the absence of any commercial or financial relationships that could be construed as a potential conflict of interest.
